# R Inguinal/R Scrotum Extramammary Paget's Disease with Diffuse Spine Metastasis Complicated by Microangiopathic Hemolytic Anemia

**DOI:** 10.1155/2018/9764049

**Published:** 2018-04-01

**Authors:** Alay Mansurov, Eric Christenson

**Affiliations:** ^1^Department of Internal Medicine, Advocate Illinois Masonic Medical Center, Chicago, IL, USA; ^2^Section of Medical Oncology, Sidney Kimmel Comprehensive Cancer Center at John Hopkins Hospital, Baltimore, MD, USA

## Abstract

A 47-year-old male presented with a groin lesion in 2011. Initial treatment with antifungals and vinegar was unsuccessful. In 2016, biopsy of this lesion was pursued with diagnosis of extramammary Paget's disease (EMPD). Prior to the scheduled excision, he developed constant lower back pain with radiation to his lower extremities. MRI confirmed vertebral metastasis. Despite surgical and radiation therapy, his back pain progressed, and repeat imaging showed epidural spread of tumor in the thoracic spine. Later, the patient was admitted to the hospital due to severe anemia and thrombocytopenia. Further work-up confirmed the diagnosis of microangiopathic hemolytic anemia (MAHA). As we know, there are only few reports of spinal metastases in patients with EMPD. To the best of our knowledge, this is the first case report of EMPD complicated by MAHA.

## 1. Introduction

Extramammary Paget's disease (EMPD) is a rare oncologic entity, best described as adenocarcinoma of the apocrine glands. Hence, it can arise in the axilla, vulva, perineum, penis, and scrotum. The most common location is vulva, accounting for 65%, followed by perianal (20%) and penis/scrotum (14%) [1]. EMPD usually presents as erosive, erythematous, circinate, or eczematous lesion over the skin. Sometimes patients complain of burning, pruritus, edema, irritation, pain, or even bleeding [2]. Though EMPD progresses slowly, it can present in advanced stage due to its nonspecific presentation [3]. It is important to keep in mind that EMPD can be secondary due to underlying malignancy. Secondary disease accounts for 10–30% of all EMPD cases [4]. Prognosis depends on stage: excellent for patients with localized lesion and poor for patients with metastatic disease [2].

In this paper, we want to share a case of R inguinal/R scrotum EMPD with diffuse spine metastasis complicated by microangiopathic hemolytic anemia (MAHA).

## 2. Case Report

**Figure 1:**
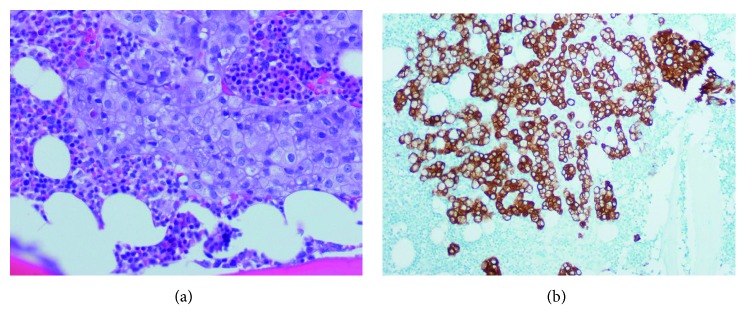
Groin skin. (a) Hematoxylin and eosin: within the dermis, there are clusters of large tumor cells with pleomorphic nuclei. (b) Cytokeratin stain highlights the tumor cells.

**Figure 2:**
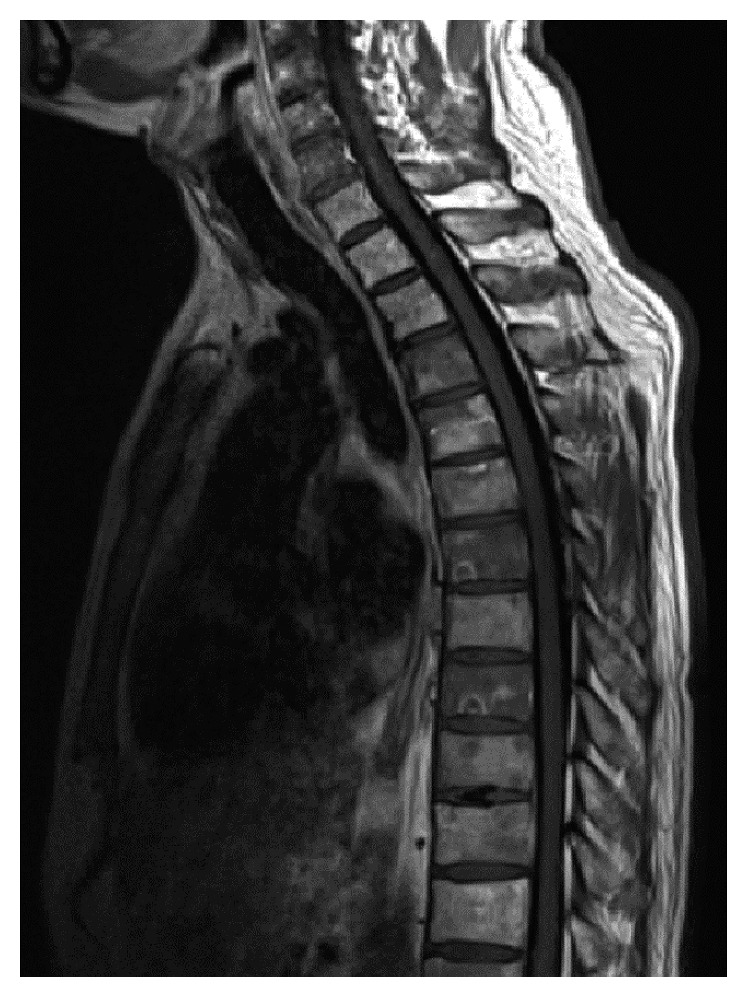
MRI of the thoracic and lumbar spine: compression vertebral fracture at L4 with retropulsion of the posterior vertebral body causing severe spinal stenosis. Extraosseous extension of tumor into ventral epidural space at T12.

A 47-year-old Asian man with a past medical history of hepatitis B on tenofovir presented with a groin lesion in 2011. Initially, antifungals and vinegar were used without substantial improvement. A biopsy was performed in 2016 and revealed EMPD (Figures 1(a) and 1(b)). Prior to the scheduled excision, the patient developed constant lower back pain with radiation to his legs, which was later accompanied by numbness. MRI of the spine showed compression vertebral fracture with severe spinal stenosis and extraosseous extension of tumor into ventral epidural space at T12 (Figure 2). The patient's symptoms progressed despite surgical intervention, conventional radiation therapy to T12 lesion, and stereotactic body radiation therapy to L4 lesion. Repeat imaging showed epidural spread of tumor in the thoracic spine at the levels of T5-T6 and T7–T10. Computed tomography of the chest, abdomen, and pelvis showed only mild right external iliac and right inguinal lymphadenopathy. No other masses or lesions were identified.

Later, the patient was admitted to the hospital due to progressive shortness of breath and fatigue. At the time of presentation, his hemoglobin was 3.8 g/dl and platelet counts were 12 × 10^9^ per liter. After 4 units of packed red blood cells (PRBCs), hemoglobin initially increased to 8 g/dl but then decreased again to 5.8 g/dl. Peripheral blood smear showed a normochromic, normocytic anemia with schistocytes, polychromasia, and no spherocytes. Additional laboratory tests were performed and confirmed diagnosis of microangiopathic hemolytic anemia (MAHA) (Table 1).

MAHA was thought to be secondary to an autoimmune phenomenon produced by the underlying cancer. Therapy with prednisone 60 mg daily for 4 weeks with further taper was initiated. Decision was made to start patient on carboplatin and paclitaxel despite severe anemia and thrombocytopenia. We were hoping that this would positively impact his transfusion requirements and disease process. After 2 cycles, repeat imaging demonstrated stable to improving disease. This prompted enthusiasm for continuing chemotherapy although no change in transfusion requirements was observed. After an additional two cycles, new imaging studies demonstrated worsening metastatic burden within the liver. Considering this finding, chemotherapy was terminated. The patient was continued to be supportively managed with PRBC and platelet transfusions.

One month later, the patient presented to the Emergency Department with dyspnea and hemoptysis. Laboratory tests were notable for hemoglobin of 4.5 g/dl, platelet counts of 11 × 10^9^ per liter, and lactic acid of 9.3 mmol/L. Unfortunately, the patient passed away after conservative management.

## 3. Discussion

MAHA in oncologic patients can be induced by cancer itself or by chemotherapy. Cancer is thought to cause MAHA by bone marrow involvement and/or systemic microvascular metastases [5]. Chemotherapy-induced MAHA occurs due to dose-dependent toxicity or drug-dependent antibody development. Distinguishing these etiologies from TTP in adults and HUS in children is essential for appropriate intervention.

It is well known that measurement of ADAMTS13 is useful for the diagnosis of TTP and administration of plasma exchange. Our patient had normal ADAMTS13 activity which excluded the underlying TTP. No usage of chemotherapy prior to development of severe anemia excludes chemotherapy-induced MAHA. These made diagnosis of cancer-induced MAHA more credible. Hence, treating underlying cancer was the only option to control MAHA in our patient. Though, repeat imaging after two cycles of carboplatin and paclitaxel demonstrated stable to improving disease, later, it progressed despite the treatment. Unfortunately, cancer-induced MAHA carries an unfavorable prognosis [5].

EMPD is a difficult diagnostic entity as a consequence of its nonspecific presentation. As a result, it can present as advanced disease. Distant metastases are rare and carry a poor prognosis.

Approximately 25% of EMPD is associated with an underlying malignancy; however, this percentage is substantially lower in lesions present in the genital area (4–7%) [6]. In a cohort of the 19 patients with genital EMPD in South Korea, only three of the patients were noted to have an underlying malignancy (colorectal, cholangiocarcinoma, and parotid gland, resp.) [7]. In our patient, computed tomography of the chest, abdomen, and pelvis was negative for the underlying malignancy. Colonoscopy was postponed, given that genital disease is far less likely to be associated with GI malignancy compared to the perianal area [6].

There are few case reports describing EMPD with skeletal metastases [8]. Given lytic nature of bone metastases, compression fractures are common. It is crucial to exclude spine pathology in EMPD patients who complain of new back pain.

Our patient was enrolled in Phase II study of postoperative stereotactic radiosurgery for solid tumor spine metastases and was treated with carboplatin and paclitaxel without success. Multiple chemotherapy regimens are used in different centers, and there is no gold-standard treatment for metastatic EMPD. Thus, early diagnosis is crucial to prevent unfavorable outcome.

## Figures and Tables

**Table 1:** Laboratory test results.

Laboratory test	Result
ADAMTS13	57%
ADAMSTS13 inhibitor	16% inhibition
Absolute reticulocyte count	245 K/m^3^
Indirect bilirubin	2.4 mg/L
Lactate dehydrogenase	950 U/L
Immature platelet fraction	21.4%
Direct Coombs test	Negative
Fibrinogen level	312 mg/dL
INR	1.1
aPTT	18.6 seconds
